# The Relationship Between Kinesiophobia and Psychological Readiness to Return to Sport After Lateral Ankle Ligament Surgery

**DOI:** 10.3390/jcm15218460

**Published:** 2026-10-30

**Authors:** Mohamad K. Moussa, Olivier Ucay, Eugénie Valentin, Emmanuel Verhaeghe, Olivier Grimaud, Pierre Garrigou, Gregoire Rougereau, Eric Laboute, Alexandre Hardy

**Affiliations:** 1 La Pitié-Salpêtrière Hospital, AP-HP, 75005 Paris, France; 2 Clinique du Sport, 75005 Paris, France; o.ucay@ramsaygds.fr (O.U.);; 3 Centre Européen de Rééducation du Sportif, 40130 Capbreton, France

**Keywords:** kinesiophobia, psychological readiness, return to sport, lateral ankle ligament reconstruction, rehabilitation

## Abstract

**Background/Objectives:** Fear of reinjury and impaired psychological readiness frequently impede return to sport following lateral ankle ligament surgery. This study aimed to analyze the correlation between kinesiophobia and psychological readiness to return to sport before and after intensive rehabilitation in competitive athletes following lateral ankle ligament repair or reconstruction (LALRR). **Methods:** A prospective longitudinal pre-post observational study was conducted at a specialized sports rehabilitation center between January 2021 and January 2024. Forty-eight competitive athletes (Tegner score ≥ 7) who underwent LALRR completed a 3-week inpatient rehabilitation program (7 h daily). Kinesiophobia was evaluated using the Tampa Scale of Kinesiophobia (TSK-17, TSK-13, TSK-11, and subscales), and psychological readiness was measured using the Ankle Ligament Reconstruction–Return to Sport after Injury (ALR-RSI) score at admission and discharge. **Results:** Cross-sectional analysis demonstrated weak to moderate negative correlations between ALR-RSI and TSK versions at baseline (rho = −0.28 to −0.43) and at discharge (rho = −0.20 to −0.60; TSK−13: rho = −0.60, 95% CI [−0.77, −0.34], *p* < 0.0001). Following rehabilitation, ALR-RSI scores significantly improved from 69.8 ± 17.3 to 76.1 ± 17.6 (*p* = 0.009), while TSK-17 scores decreased from 36.9 ± 7.8 to 33.2 ± 7.3 (*p* = 0.0001). However, the longitudinal correlation between individual change scores was weak and non-significant (rho = −0.15, 95% CI [−0.41, 0.11], *p* = 0.32). **Conclusions**: Kinesiophobia and psychological readiness exhibit weak to moderate inverse correlations cross-sectionally. However, no statistically significant association was detected between changes in kinesiophobia and psychological readiness in this sample (rho = −0.15, *p* = 0.32). These metrics capture distinct psychological aspects, and both should be monitored independently when assessing athletes prior to sports resumption.

## 1. Introduction

Chronic ankle instability (CAI) is a common condition that significantly impacts an athlete’s ability to return to sport and perform at their optimal level [1,2,3,4,5]. It is often characterized by recurrent episodes of ankle sprains, persistent pain, and a sense of instability, which can lead to long-term functional impairments [4,5,6,7]. Surgical intervention, such as lateral ankle ligament reconstruction (LALR), is frequently required for athletes who fail to respond to conservative treatments [4,8,9,10]. However, even after surgical repair, psychological factors such as fear of movement (kinesiophobia), fear of reinjury, and apprehension can hinder recovery and delay the return to competitive sports [11,12,13,14,15]. While surgical stabilization—such as the modified Broström-Gould anatomical repair or anatomical tendon reconstruction—reliably restores mechanical joint stability in over 85% to 90% of athletes [4,8,9,10], mechanical restoration alone does not automatically equate to a successful or timely resumption of competitive sports [11,12,13,14,15]. Persistent functional limitations are frequently driven by maladaptive psychological responses that delay full athletic clearance [11,12,13,14,15].

Kinesiophobia, as measured by the Tampa Scale of Kinesiophobia (TSK) [16], has been shown to negatively impact functional outcomes across a range of musculoskeletal conditions [11,12,15,17]. Studies have demonstrated its association with patient-reported outcome measures, poor postural control, decreased physical activity levels, and reduced quality of life [11,12,15,17]. On the other hand, psychological readiness to return to sport, assessed using the Ankle Ligament Reconstruction–Return to Sport after Injury (ALR-RSI) score, represents another critical factor in recovery, addressing the athlete’s confidence and emotional preparedness to resume sport-specific activities [13,14].

While kinesiophobia has been extensively linked to physical function and many patient-reported outcome measures [11,12,15,17], its relationship with psychological readiness to return to sport, as assessed by the ALR-RSI, is less clear and has been insufficiently explored. Although kinesiophobia (fear of movement/reinjury) and psychological readiness (confidence, emotional readiness, and risk appraisal) overlap, they capture distinct constructs that may evolve asynchronously during rehabilitation. To our knowledge, this relationship has not previously been investigated in athletes following lateral ankle ligament surgery.

The aim of this study was to analyze the correlation between kinesiophobia, as measured by the TSK, and psychological readiness to return to sport, as measured by the ALR-RSI, before and after a rehabilitation program following lateral ankle ligament reconstruction or repair (LARR). Additionally, the study aimed to evaluate the observed gains for each score and their clinical relevance.

The study hypothesized that, following three weeks of rehabilitation after LALRR, there would be a significant increase in psychological readiness (ALR-RSI score) and a significant decrease in kinesiophobia (TSK score). However, these changes were expected to be uncorrelated, as the two scales assess distinct psychological constructs.

## 2. Material and Methods

### 2.1. Study Design and Inclusions

This was a prospective longitudinal pre-post observational study targeting athletes with chronic ankle instability who had undergone LALRR. The study was conducted at a specialized sports physical therapy center between January 2021 and January 2024.

Inclusion criteria required participants to be over 18 years of age, actively engaged in competitive sports (Tegner activity scale ≥ 7), and referred for physical therapy following LALRR. Patients were excluded if they were not involved in competitive sports (Tegner activity scale < 7), had undergone revision surgery, presented with osteochondral lesions of the talus requiring standalone cartilage procedures without ligament repair or reconstruction, had isolated tibiofibular joint injuries, or refused to participate.

The study was approved by the institutional review board (IRB00010835), and written informed consent was obtained from participants before inclusion in the study.

### 2.2. Indication and Rehabilitation Protocol

Surgery was indicated for patients experiencing pain and ankle instability despite completing an initial course of conservative management and presenting with ankle laxity among clinical testings. Reconstruction was preferred to reparation for individuals with advanced ligament damage classified as Stage 3 or 4 (thin or absent ATFL) based on the French Society of Arthroscopy criteria [18]. Surgical intervention was performed by experienced orthopedic sports medicine specialists. Anatomic repair using the modified Broström-Gould procedure was performed when viable native ligament tissue was present. In cases of chronic, severe tissue attenuation or defect (Stage 3 or 4 anterior talofibular ligament lesions per the French Society of Arthroscopy criteria), anatomic reconstruction was executed using either autologous gracilis tendon or tendon allograft secured with interference screws and suture anchors. Associated intra-articular pathologies (e.g., superficial chondral lesions or syndesmotic laxity) were addressed concurrently as indicated in Table 1. Following initial postoperative immobilization and progressive weight-bearing clearance, all participants completed a standardized, intensive 3-week inpatient rehabilitation program at a specialized sports center (7 h daily, 5 days per week).

The rehabilitation program was delivered in a specialized sports rehabilitation center over three consecutive weeks. Participants attended the center daily for a 7 h period under the supervision of physiotherapists and strength and conditioning coaches. Each day combined active joint mobilization, progressive resistance strengthening (focusing on peroneal and intrinsic foot musculature), neuromuscular training, physical reconditioning, and progressive sport-specific functional tasks. Exercise dosage and progression were individualized and criterion-based rather than determined solely by time. Progression to higher training loads, dynamic plyometrics, and running (initiated on an Alter-G anti-gravity treadmill and advanced to outdoor running) required painless full weight-bearing, symmetrical active range of motion, satisfactory movement quality, and absence of reactive joint effusion. Each athlete underwent a weekly physician review to validate the progression plan based on clinical examination and functional test tolerance.

### 2.3. Outcome Measures and Analysis

The primary analysis was the correlation between the TSK and the ALR-RSI score.

The French version of the ALR-RSI was used to assess psychological readiness to return to sport [13,14]. This 12-item questionnaire measured three psychological domains: emotions, performance confidence, and risk appraisal. Each item was scored from 0 to 10, and the total score was calculated by summing the responses, dividing by 1.2, and expressing the result as a percentage. Higher scores corresponded to more positive psychological responses.

The TSK, a validated tool designed to assess fear of movement or reinjury, was analyzed in three versions and two subscales: the original full scale (TSK-17) [19], a shortened 13-item version (TSK-13) [16], and the more concise 11-item version (TSK-11) [20]. Higher scores on all versions indicated greater levels of kinesiophobia. The TSK-17, with a total score range of 17–68, was used to provide a comprehensive measure of kinesiophobia with a threshold at 40. The TSK-13, scoring between 13 and 52, retained essential items while reducing length. The TSK-11, with a range of 11–44, was developed to capture the core elements of kinesiophobia with minimal burden on participants. The TSK included two subscales: avoidance and somatic focus. The avoidance subscale consisted of items measuring behavioral tendencies to avoid physical activity due to fear of pain or injury; the threshold is 15. The somatic focus subscale consisted of items reflecting cognitive beliefs that pain indicated severe underlying medical issues; the threshold is 10. Scores were collected at two time points: at the start (entry) and conclusion (exit) of the 3-week rehabilitation program.

The secondary analysis evaluated the progression of these scores after the rehabilitation session, comparing the magnitude of changes between the measures. The changes were reported as a difference in mean as well as a difference in percentage of patients crossing the thresholds of kinesiophobia set at 40 for TSK, 15 for the avoidance subscale, and 10 for the somatic focus subscale [21].

### 2.4. Data Collection

Data were obtained from patient records and self-reported questionnaires completed during rehabilitation sessions. Scores were collected prospectively, with patients filling out the questionnaires under the supervision of a medical physician to ensure accuracy and consistency in responses. The variables collected included the previously mentioned outcome measures (TSK-17, TSK-13, TSK-11, SF, AA, and the ALR-RSI score), and demographic information such as age, gender, and injury type. The validated French version of the TSK was administered under medical supervision. All participants completed the full 17-item instrument, and scores for the shortened versions (TSK-13, scoring 13–52; TSK-11, scoring 11–44) and subscales (avoidance and somatic focus) were calculated by extracting their respective validated item subsets.

### 2.5. Participants and Sample Size

**Figure 1:**
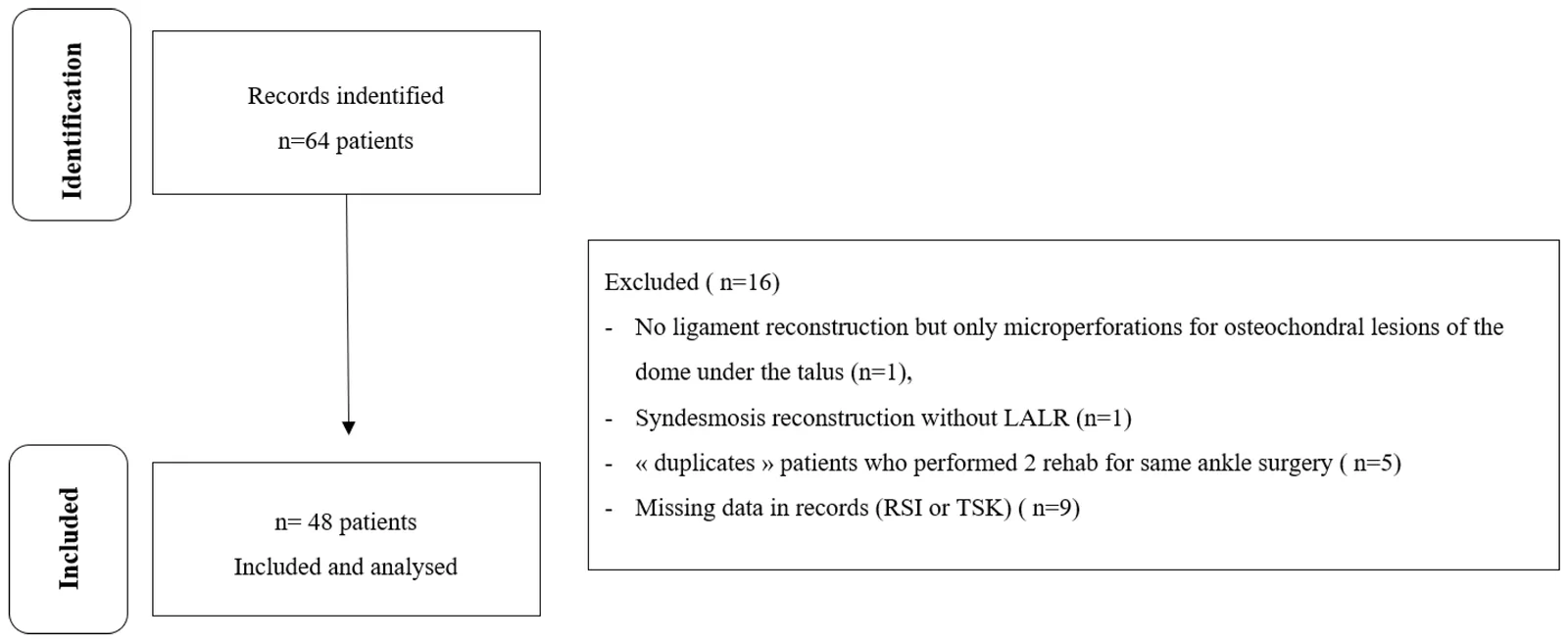
Flowchart.

Of the 64 records screened, 9 patients with incomplete questionnaire pairs were excluded, resulting in a final cohort of 48 athletes who completed all baseline and exit assessments (Figure 1).

In this study, a sample size of 48 participants produces a two-sided 95% confidence interval with a width smaller than 0.35 when the estimate of Spearman’s rank correlation is above 0.70. The initial sample size calculation was based on an expected strong correlation (rho ≥ 0.70) with a 95% confidence interval width < 0.35 (Figure 1). While lower observed correlations inevitably result in wider confidence intervals around point estimates, post hoc power analysis confirmed that our sample of 48 patients retained a statistical power exceeding 0.95 to detect correlation coefficients of rho ≥ 0.50 at alpha = 0.05, providing adequate power for our primary findings.

### 2.6. Statistical Analysis

Descriptive statistics were computed using frequencies and percentages for categorical variables and mean (standard deviation) or median and interquartile range (IQR) for continuous variables, depending on data distribution. Normality of continuous variables was assessed using the Shapiro–Wilk test.

A Spearman correlation matrix was used to assess the relationships between different psychological measures at entry and at the end of rehabilitation. Spearman’s rank correlation coefficient (ρ) was interpreted as follows: ρ < 0.10: no correlation; ρ = 0.10–0.39: weak correlation; ρ = 0.40–0.69: moderate correlation and ρ ≥ 0.70: strong correlation.

Changes in psychological scores between the beginning and end of rehabilitation were assessed using the Wilcoxon signed-rank test, appropriate for paired and non-normally distributed data. Changes in the proportion of patients below each kinesiophobia threshold between entry and exit were compared using the exact McNemar test for paired binary data.

All analyses were performed using R software (version 4.2), with a significance threshold set at *p* < 0.05.

## 3. Results

### 3.1. Patient Characteristics (Table 1)

Eighteen women and 30 men, aged 28.8 ± 9.78 years old, were included; patients began their 3-week rehabilitation program at 88.2 ± 58.9 days after surgery (Table 1).

### 3.2. Primary Analysis

**Figure 2:**
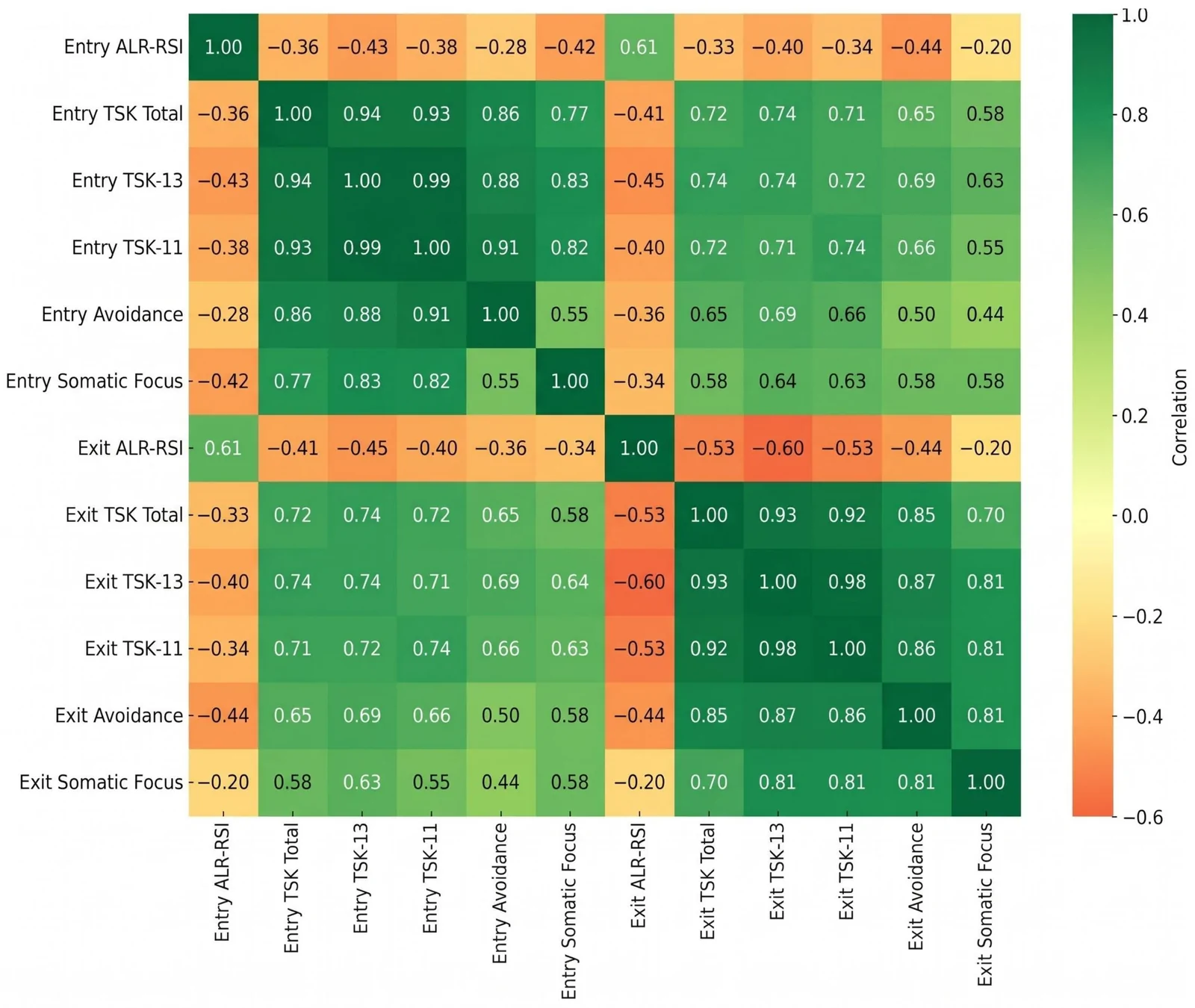
Correlation matrix between scores.

At entry, the ALR-RSI score showed mild to moderate negative correlations with the TSK scores across all versions (TSK-17, TSK-13, TSK-11, and the somatic focus and avoidance subscales), with correlation values ranging from rho = −0.28 to −0.43, with the strongest negative correlation observed between the ALR-RSI and the TSK-13 (rho = −0.43, 95% CI [−0.63, −0.19], *p* = 0.002). At exit, the correlations ranged from rho = −0.20 to −0.60, with the strongest negative correlation observed between the ALR-RSI and the TSK-13 (rho = −0.60, 95% CI [−0.77, −0.34], *p* < 0.0001) (Figure 2). 

### 3.3. Changes in TSK and ALR-RSI After Rehabilitation

The mean ALR-RSI score increased from 69.8 (SD 17.3) at entry to 76.1 (SD 17.6) at exit, representing an absolute mean improvement of 6.3 points (relative increase of 9.0%; *p* = 0.009). The mean TSK-17 score decreased from 36.9 (SD 7.8) to 33.2 (SD 7.3), reflecting an absolute reduction of 3.7 points on the 68-point scale (relative reduction of 10.0%; *p* = 0.0001) (Table 2).

The correlation between changes in TSK and ALR-RSI scores was weak and negative (rho = −0.15, 95% CI −0.41; 0.11, *p* = 0.32).

After rehabilitation, the percentage of patients scoring below the high-kinesiophobia threshold (TSK-17 < 40) increased significantly from 58.3% (28/48) to 81.3% (39/48) (*p* = 0.003). In contrast, changes in the proportions of participants falling below the subscale thresholds did not reach statistical significance: the proportion below the avoidance threshold (< 15) rose from 45.8% (22/48) to 60.4% (29/48) (exact McNemar *p* = 0.06), and the proportion below the somatic focus threshold (< 10) increased from 52.1% (25/48) to 66.7% (32/48) (exact McNemar *p* = 0.12; Table 3).

## 4. Discussion

The main finding of this study was a weak to moderate negative correlation between kinesiophobia and psychological readiness to return to sport both before and after rehabilitation.

To our knowledge, the relationship between kinesiophobia and psychological readiness to return to sport specifically using the ALR-RSI has not been previously investigated in patients undergoing lateral ankle ligament surgery. Existing research has primarily focused on correlations between the TSK and other patient-reported outcome measures or functional parameters [11,12,15,16,17]. For example, Watanabe et al. demonstrated a moderate negative correlation between the TSK and ankle function measures, such as the FAAM Sports subscale (*r* = −0.5, *p* = 0.005), while also noting weak correlations with quality-of-life measures such as the SF-36 physical component summary [15]. Similarly, Alshahrani et al. found that kinesiophobia significantly predicted errors in ankle joint position sense and postural sway in individuals with functional ankle instability, suggesting its direct impact on neuromuscular control [11]. Han et al. further observed that individuals with high levels of kinesiophobia relied more on visual input for balance, indicating a greater reliance on compensatory strategies in postural control [12]. Lastly, Walankar et al. reported that kinesiophobia was associated with poorer physical function and reduced quality of life, emphasizing the need to address this psychological barrier during rehabilitation [17].

It is essential to distinguish the static cross-sectional correlations observed at admission and discharge from the correlation between score changes over time. While static scores demonstrated weak to moderate negative correlations at both entry and exit, no statistically significant association was detected between changes in kinesiophobia and psychological readiness in this sample (rho = −0.15, 95% CI [−0.41, 0.11], *p* = 0.32). This indicates that individual reductions in kinesiophobia did not correlate with concomitant gains in psychological readiness over this 3-week interval.

The findings of this study align with the results observed in knee injuries, particularly in anterior cruciate ligament (ACL) research [22,23]. A study by Ohji et al. demonstrated that kinesiophobia, as measured by the TSK, was negatively associated with psychological readiness to return to sport in patients awaiting ACL reconstruction, with a weak to moderate negative correlation (*r* = −0.305, *p* = 0.02). Notably, multiple regression analysis revealed that TSK score was the only factor significantly associated with psychological readiness to return to sport (*p* = 0.002) [23].

This study demonstrated that a structured three-week rehabilitation program following LALRR led to significant improvements in both psychological readiness to return to sport and a reduction in kinesiophobia. These findings suggest that fear of movement and return-to-sport readiness reflect complementary, non-redundant psychological constructs that warrant individual consideration during postoperative rehabilitation. Importantly, while total continuous scores on both the ALR-RSI and TSK-17 demonstrated statistically significant improvements, and the proportion of patients classified below the overarching TSK-17 cutoff increased significantly (*p* = 0.003), categorical shifts below the individual subscale thresholds for avoidance (*p* = 0.06) and somatic focus (*p* = 0.12) were not statistically significant. This indicates that while intensive rehabilitation consistently reduces overall kinesiophobia burden, reductions across specific cognitive subdomains—such as somatic symptom focus and fear-avoidance beliefs—exhibit greater individual variability and did not achieve uniform statistical significance in this cohort. However, because this study lacked an unexposed control group, the observed improvements in kinesiophobia and psychological readiness cannot be definitively attributed to the rehabilitation program alone; natural psychological adaptation, surgical healing, and the passage of time remain potential confounders. Previous studies have shown that rehabilitation interventions can influence psychological readiness, such as Picot et al., who demonstrated that taping and bracing significantly improved the Ankle-GO score by increasing his ALR-RSI component, despite having no effect on functional performance [24].

This study has several limitations. First, the lack of an unexposed control group prevents establishing direct causality between the rehabilitation protocol and the psychological improvements observed. Second, our sample size, while powered post hoc (>0.95) for primary moderate correlations, limited subgroup comparisons across surgical techniques. Third, the 3-week follow-up was relatively short and did not track actual return-to-sport rates or long-term reinjury incidence, leaving the ultimate prognostic utility of these score changes to be determined. Additionally, patients undergoing both anatomic repair and tendon reconstruction were analyzed collectively, and the interval between surgery and initiation of rehabilitation varied across participants (mean 88.2 ± 58.9 days). Although all athletes completed the same standardized 3-week inpatient protocol, differences in surgical invasiveness and postoperative tissue healing stages may have influenced baseline psychological scores and should be considered when interpreting these findings. It is also worth noting that the TSK-13, TSK-11, and the two subscales are derived from overlapping items of the TSK-17; therefore, the secondary analyses should be interpreted with caution.

## 5. Conclusions

In athletes recovering from lateral ankle ligament repair or reconstruction, kinesiophobia and psychological readiness to return to sport exhibit weak to moderate inverse correlations cross-sectionally. However, no statistically significant association was detected between changes in kinesiophobia and psychological readiness in this sample (rho = −0.15, 95% CI [−0.41, 0.11], *p* = 0.32). These findings suggest that fear of movement and return-to-sport confidence assess different aspects of the athlete’s psychological profile during recovery. Clinicians should evaluate both dimensions individually rather than assuming improvement in one guarantees progress in the other. This study is limited by the lack of an unexposed control cohort, heterogeneity in surgical techniques and time-from-surgery to rehabilitation entry, and lack of long-term tracking to actual return-to-competition or subsequent reinjury. Future multicenter prospective studies should track long-term functional and psychological milestones up to 2 years postoperatively, evaluate targeted cognitive-behavioral adjuncts alongside physical conditioning, and establish domain-specific cutoffs to optimize psychological readiness prior to sports clearance.

## Figures and Tables

**Table 1:** ***Patient characteristics.*** Socio-demographic and clinical characteristics of patients (*n* = 48).

Characteristics	Total (*n* = 48)
Age, years	28.8 ± 9.78
**Sex**	
Female, *n* (%)	18 (37.5)
Male, *n* (%)	30 (62.5)
**Sport Level** *n* (%)	
Physical professions (e.g., military)	18 (37.5%)
Regional	15 (31.3%)
National	13 (27.1%)
International	2 (4.2%)
**Sports types *n* (%)**	
Pivot contact sport	22 (45.8%)
Other	26 (54.2%)
**Technique**	
Broström-Gould	28 (58.3%)
Anatomic reconstruction with gracilis	17 (35.4%)
Anatomic reconstruction with Allograft	2 (4.3%)
Hemi-Castaing	1 (2.2%)
**Associated procedure**	
MCL suture	2
OLT	2
Syndesmosis reconstruction	1

**Table 2:** Changes in ALR-RSI and TSK before and after the rehabilitation program.

	Score at Entry	Score at Exit	Differences	% Change	*p*-Value
**ALR-RSI (/100)**					
**median (IQR)**	68.3 (60.0, 84.6)	79.6 (68.3, 88.3)	4.2 (−4.2, 15.8)	+6.1%	0.009
**mean (sd)**	69.8 (17.3)	76.1 (17.6)	6.2 (14.3)	+9.0%	0.009
**TSK-17 score (/68)**					
**median (IQR)**	35.0 (31.0, 43.0)	32.5 (29.0, 38.5)	−4.5 (−7.0, 0.5)	−12.9%	0.0001
**mean (sd)**	36.9 (7.8)	33.2 (7.3)	−3.7 (5.8)	−10.0%	0.0001

Note: Relative change (%) is calculated as ((Exit − Entry)/Entry) × 100.

**Table 3:** Changes in percentage of patients crossing the kinesiophobia threshold.

	Before Rehabilitation	After Rehabilitation	*p*-Value
**Score TSK-17**			0.003
**<40**	28 (58.3%)	39 (81.3%)	
**≥40**	20 (41.7%)	9 (18.8%)	
**The avoidance subscale**			0.06
**<15**	22 (45.8%)	29 (60.4%)	
**≥15**	26 (54.2%)	19 (39.6%)	
**The somatic focus subscale**			0.12
**<10**	25 (52.1%)	32 (66.7%)	
**≥10**	23 (47.9%)	16 (33.3%)	

## Data Availability

The data presented in this study are available on reasonable request from the corresponding author.
